# Sleeping and Ranging Behavior of the Sambirano Mouse Lemur, *Microcebus sambiranensis*

**DOI:** 10.1007/s10764-017-9997-2

**Published:** 2017-10-28

**Authors:** Dan Hending, Grainne McCabe, Marc Holderied

**Affiliations:** 1 School of Biological Sciences, The University of Bristol, Bristol, BS8 1TH UK; 2 Bristol Zoological Society, Clifton, Bristol, BS8 3HA UK

**Keywords:** Home range, Lemur, *Microcebus Sambiranensis*, Sleeping ecology

## Abstract

Primates require secure sleeping sites for periods of rest, but despite their importance, the characteristics of desired sleeping sites are poorly known. Here we investigated the sleeping ecology of a radio-collared population of the Sambirano mouse lemur, *Microcebus sambiranensis*, during the nonreproductive season in the Anabohazo forest, northwestern Madagascar. We also investigated their ranging behavior and examined the spatial distribution of sleeping sites within the home ranges of the collared individuals. We took measurements of the sleeping tree’s physical characteristics and recorded the number of collared individuals using each sleeping site. We found that *M. sambiranensis* generally use foliage sleeping sites more frequently than tree holes and individuals slept more frequently in densely foliated trees than in sparsely foliated trees, often alone. We observed no significant differences in home range size or nightly travel distance between males and females; however, home ranges were smaller than those described for other mouse lemur species. Finally, we found that *M. sambiranensis* sleep peripherally and forage centrally within their home ranges, a behavior not previously described for mouse lemurs. Our results indicate profound differences in the social organization between *M. sambiranensis* and other mouse lemur species described in the literature, suggesting species-specificity in mouse lemur ecology. Understanding the sleeping ecology and ranging behavior of mouse lemurs is of great importance to their conservation, as these data facilitate the planning of long-term reforestation, habitat management, and population assessment.

## Introduction

Secure sleeping sites can be critical for the survival of primates, as they are highly vulnerable to predation (Radespiel *et al.* 2003; Rode *et al.* 2013; Seiler *et al.* 2013a). Primates have been observed to use a range of different sleeping site types such as tree holes and cavities, foliage nests, tree crowns, rocky cliff faces, constructed nests or dens, and even man-made structures (Lutermann *et al.* 2010; Semel and Ferguson 2012). As well as possibly providing a means of protection from predators, tree holes and enclosed foliage nests provide thermal insulation for nocturnal primate species that enter daily or seasonal torpor (Schmid 1998). Sleeping sites and their availability have also been suggested to influence ranging behavior (Anderson 2000). Many primate taxa such as lemurs (Lutermann *et al.* 2010; Rode *et al.* 2013; Seiler *et al.* 2013a; Weidt *et al.* 2004), galagos (Nash and Harcourt 1986), and lorises (Nekaris 2003; Wiens and Zitzmann 2003) use multiple sleeping sites within the boundaries of their home range. Knowledge of the physical characteristics and usage patterns of sleeping sites is limited to a small number of primate species, despite their potential importance for the survival of these animals (Lutermann *et al.* 2010).

The nocturnal lemurs of Madagascar form such a group of primates where knowledge on their sleeping site usage is limited. These are currently 75 described species in 8 genera, some of which undergo periods of torpor (Groves 2016; Louis and Lei 2016; Mittermeier *et al.* 2010). The characteristics of sleeping sites used by some of these genera are well studied. For example, sportive lemurs, such as *Lepilemur mustelinus*, *L. edwardsi*, and *L. sahamalazensis*, frequently use tree holes situated in tall trees with protective dense canopies and rarely use foliage nests (Rasoloharijaona *et al.* 2003, 2008; Seiler *et al.* 2013a). Some dwarf lemur species such as *Cheirogaleus sibreei* and *C. major* sleep exclusively in tree holes, whereas *C. crossleyi* sleeps in both tree holes and foliage nests (Blanco and Godfrey 2014; Lahann 2008). Furthermore, *C. medius* prefer large trees with well-insulated sleeping sites that provide optimal insulation properties for the individuals within to control their energy expenditure during torpor (Dausmann 2013; Dausmann and Blanco 2016). Mouse lemurs range and sleep within a social neighbourhood system, where individuals are familiar with whom they share their sleeping sites and with whom their ranges overlap, despite the lack of cohesive social groups (Atsalis 2000; Dammhahn and Kappeler 2005; Génin 2008). Mouse lemurs require sleeping sites that offer protection from predators and, in species that undergo torpor, thermoregulatory functions that provide a buffer against low temperatures (Radespiel *et al.* 1998; Schmid 1998). The sleeping ecology of 5 of the 24 currently described mouse lemur species has been thoroughly investigated: *Microcebus murinus*, *M. rufus*, *M. ravelobensis*, *M. berthae*, and *M. griseorufus*. Many of these studies have focused on the suitability of sleeping sites for daily and seasonal torpor in relevant species such as *M. murinus* and *M. rufus* (Atsalis 1999a; Perret 1998; Schmid 1998). Other studies have focused on sex-specificity in sleeping site ecology (Radespiel *et al.* 1998) and species-specific usage of sleeping sites, as well as interspecific site competition (Radespiel *et al.* 2003). Specific mouse lemur sleeping site types are well known, with some species such as *M. murinus* having a preference for tree hole sleeping sites (Radespiel *et al.* 2003; Rasoazanabary 2006), while others such as *M. berthae* have a preference for constructed vegetation sleeping sites (Schwab 2000).

The link between ranging and sleeping behaviors has not been investigated for mouse lemurs, despite the large numbers of studies on mouse lemur ranging and sleeping ecology (Dammhahn and Kappeler 2005; Radespiel 2000). Studies of ranging behavior in mouse lemurs have instead focused on home range size and their size fluctuation in relation to seasonality and food availability (Génin 2008; Schmid *et al.* 2000). These studies reveal species differences in ranging behavior (Dammhahn and Kappeler 2005; Génin 2008; Schmid 1998; Schmid *et al.* 2000). For example, *Microcebus berthae* has a relatively large, seasonally fluctuating home range of up to 4.9 ha (Dammhahn and Kappeler 2005) compared to the less fluctuating 1.8-ha home range of *M. murinus* (Pages-Feuillade 1988) and the even smaller 0.7 ha home range of *M. rufus* (Radespiel 2006). Investigation into mouse lemur ranging behavior with regard to sleeping site locations is needed to better understand the link between these two behaviors.

The Sambirano mouse lemur, *Microcebus sambiranensis*, is one of the smallest species in the *Microcebus* genus and is known to inhabit only a few small forests in northwestern Madagascar (Randriatahina *et al.* 2014; Rasoloarison *et al.* 2000). To date this species is completely unstudied and nothing is known of its sleeping behavior or ecology. It is therefore an ideal species to further investigate mouse lemur sleeping and ranging behavior. Here, we investigated the physical characteristics of the sleeping trees used by a radio-collared study population of *M. sambiranensis* and the group composition and sex-specific usage of these sleeping sites, with the overall aim of gaining an insight into the sleeping behavior social organization of this species. We focused our investigation on the physical properties of the sleeping tree, rather than the thermoregulatory properties, as it is not yet known whether *M. sambiranensis* undergoes periods of torpor. We hypothesized that *M. sambiranensis* would more frequently use sleeping sites with good concealment from predators from dense surrounding microhabitats. We predicted that *M. sambiranensis* individuals would have a sleeping site niche preference (tree holes or foliage nests), as has been observed in *M. murinus* and *M. ravelobensis* (Radespiel *et al.* 2003). In parallel, we investigated the ranging behavior and nightly travel distances of the radio-collared individuals. We predicted that there would be large overlaps in the home ranges of our study individuals, as was found in studies of *M. murinus* (Eberle and Kappeler 2002; Radespiel 2000). Finally, we linked our results of sleeping site ecology and ranging behavior to examine the spatial distribution of sleeping sites throughout the respective individuals’ home ranges.

## Methods

### Study Site

**Fig. 1:**
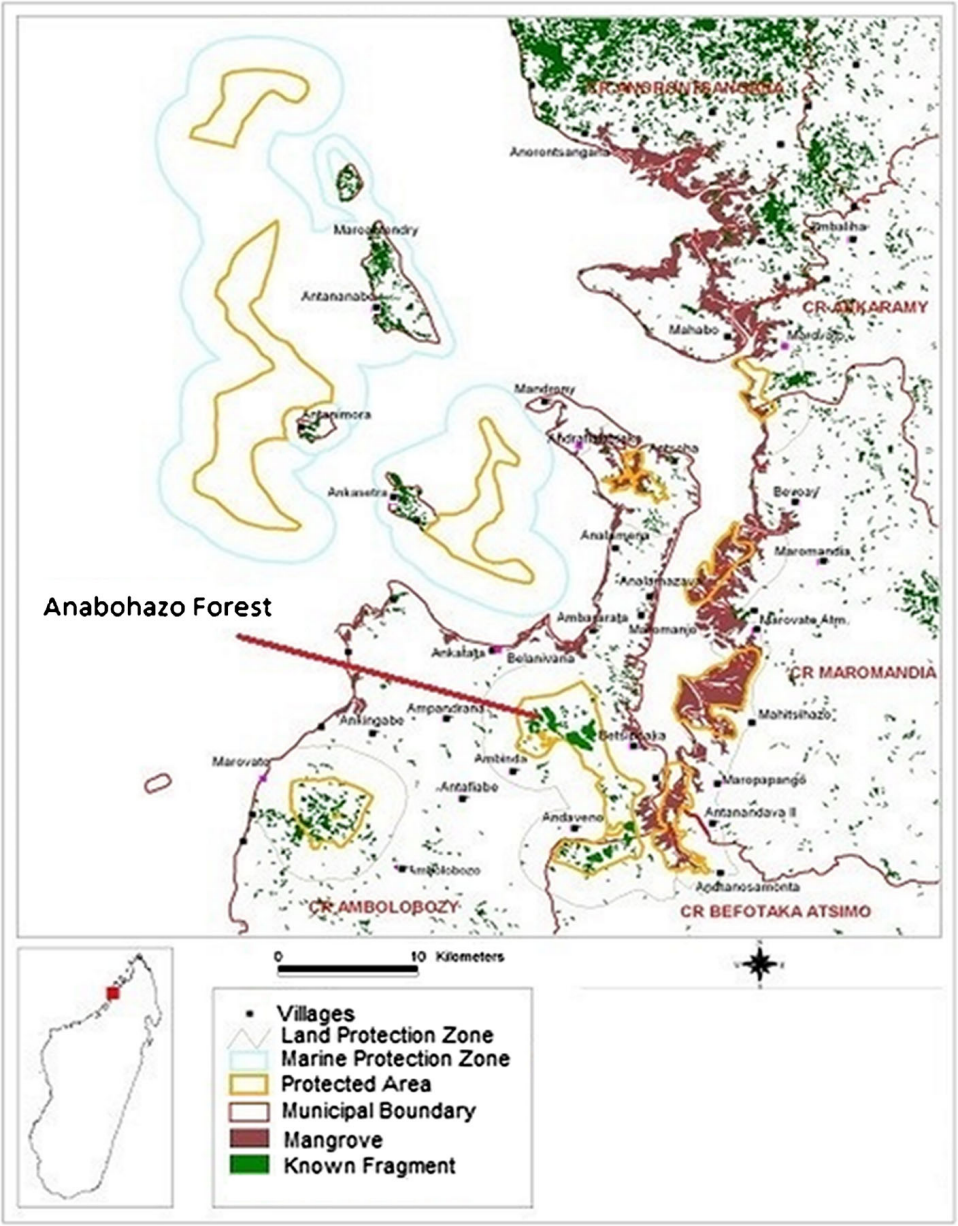
The Sahamalaza Peninsula, northwest Madagascar with the Anabohazo forest study site indicated. Adapted from USBD-WCS Madagascar (2006).

The Anabohazo Forest is located in the northeast sector (S14°19′, E47°54′) of the Sahamalaza–Iles Radama National Park (Fig. 1) in the Sofia region of northwestern Madagascar (Seiler *et al.* 2013b). Since 2001, Sahamalaza has been a UNESCO Biosphere Reserve and was designated a National Park in 2007 (Volampeno *et al.* 2015). Managed by Madagascar National Parks (MNP), the protected area of the Sahamalaza–Iles Radama National Park extends between S13°52′ and S14°27′ and E45°38′ and E47°46′ (WCS/DEC 2002). The Sahamalaza–Iles Radama National Park is located within the Sambirano domain of Madagascar, a transitional area between the rainforests of the north and the drier deciduous forests of the west (Project ZICOMA 1999). Sahamalaza has a hot and subhumid climate (Andreone *et al.* 2001) with a wet season from November until April followed by a dry season from May until October. Temperatures fluctuate around 28.0 °C throughout the year, with a maximum mean temperature of 32.0 °C, minimum mean temperature of 20.6 °C, and an extreme temperature range of 13.2 °C–39.1 °C (Schwitzer *et al.* 2007; Volampeno *et al.* 2011). Mean precipitation for the area is 1750 mm of rainfall (Project ZICOMA 1999), most of which falls in the wet season. Anabohazo is a semihumid forest, characterized by rolling hills of ca. 300–350 m a.s.l. that are intersected by small, seasonal streams (Andreone *et al.* 2001). Anabohazo has a total area of 5275 ha, the largest of the forest blocks remaining on the peninsula (Randriatahina *et al.* 2014). The vegetation is characteristic of the western dry forests of Madagascar but there are many tree species here unique to the Sambirano domain (Birkinshaw 2004). The presence of the Endangered Sambirano mouse lemur was first confirmed in the National Park in 2014 (Randriatahina *et al.* 2014).

### Collaring

We captured eight *Microcebus sambiranensis* individuals using live traps (LFG Folding Trap, Sherman, Tallahassee, FL, USA) under the supervision of a team of qualified, experienced veterinarians over a 4-day period at the start of the investigation, March 23, 2015–March 26, 2015. We set 10 traps nightly at 18:00 h and checked them every hour until 02:00 h. We logged GPS waypoints of the capture site trees. The veterinarian team anesthetized the captured individuals using a 10 mg/kg IM dose of Telazol® anesthetic, identified their sex, and weighed them to determine if they were suitable for collaring (five males and three females, weights 19.5–47.0 g). The veterinarians noted no signs of swelling, estrus, lactation, or pregnancy in the captured females, suggesting that our study period was not during the reproductive or gestation period of *M. sambiranensis*. The veterinarians then collared the individuals with TW4 PiP lightweight collars (BioTrack, Wareham, UK; frequency range 150.061–150.508, weight 1.5 g). The veterinarian team secured the collars around the individuals’ necks using a zip tie, tightening them carefully to ensure that accidental asphyxiation did not occur and then placed the individuals in bags to recover from the effects of the anesthesia. Any remaining exposed length of cable tie was cut off using scissors. Once fully recovered, we released the collared individuals back at the tree in which they were captured at the beginning of the subsequent night of the capture. We recaptured all collared individuals at the end of the study and removed their collars.

### Sleeping Site Characterization

We located the collared *Microcebus sambiranensis* individuals daily using a portable radio receiver (Regal 2000) with a two-element antenna (both Titley Scientific, Columbia, MO, USA) during daylight hours over 30 days, March 28, 15–April 26, 2015, to reveal the location of their sleeping site tree. We identified and described the tree species of the sleeping site tree based on the existing literature on Sambirano domain vegetation (Schatz 2001; Seiler 2012; Van den Abeele 2014). We characterized the sleeping site as either a tree hole or a foliage nest constructed by the lemur and measured the following tree characteristics; DBH: the diameter of the trunk at breast height; tree height: the vertical distance between the upper boundary of the leaves and the trunk at ground level; bole height: the vertical distance between the lowermost level of branches and the trunk at ground level, and crown diameter at its maximum horizontal width. We measured DBH with measuring tape, and tree height and bole height to the nearest half-meter with a laser rangefinder (MasTech, Pittsburgh, PA, USA). We also used the point-centered quarter method to measure how densely vegetated the microhabitat surrounding the sleeping tree was, following Cottam and Curtis (1956). This involved measuring the distance to the nearest neighboring tree in four geographic directions surrounding the sleeping tree. We then squared the mean of these four point-to-plant distances and calculated its inverse to give us the microhabitat density in number of trees/m^2^ (n/m^2^). We recorded GPS waypoints and elevations for each sleeping site with a handheld GPS (eTrex 30, Garmin, Schaffhausen, Switzerland). We used the radio receiver to test for signals of other collared mouse lemurs at the sleeping site to confirm if the individual was sharing its sleeping site with another collared lemur. We could not check for the presence of uncollared individuals within the sleeping sites as we would have needed to further open the sleeping site by hand to visually do this, consequently disturbing the individuals within. We marked all confirmed sleeping site trees with biodegradable tape so we could distinguish between new and reused sites. If we found individuals using the same sleeping site from a previous night, we recorded this as a reuse.

### Home Ranges

We recorded home range data from March 26, 2015 to May 30, 2015, a period not thought to include the reproductive period based on a lack of observational signs of swelling and estrus in our captured individuals. We conducted 35 nocturnal follows during this period where we located a collared individual at dusk (ca. 18:00 h) when it was leaving its sleeping site using radiotelemetry. Our three-person team then followed the individual until dawn (ca. 05:00 h), when it returned to a sleeping tree (*N* = 26), or until we lost the radio signal and could not visually relocate the individual in 30 min of searching time (*N* = 9). We recorded GPS waypoints of the individual’s movements for every tree that the individual traveled through using a handheld GPS (eTrex 30, Garmin, Schaffhausen, Switzerland). We preferred this method to periodical waypoint logging, as we could not always visually locate an individual if it was hidden among dense foliage to confirm the exact tree that it was in. If we observed an individual resting for an extended period of time in one tree, we recorded only one GPS point for that tree. Additionally, we logged a GPS waypoint for each tree that we observed an individual to feed in; we labeled these as feeding trees.

We calculated the home range size of each individual (*N* = 8) in ArcGIS ArcMap 10.0 (esri, Redlands, CA, USA) using all GPS waypoints recorded in nocturnal follows and all GPS locations of sleeping sites via minimum concave polygons. We preferred the minimum concave polygon method to kernel analysis because we used two independent datasets (nocturnal follow GPS waypoints and sleeping site locations) to determine home range size. This is because most GPS locations were from nocturnal follows rather than sleeping site locations and the pooling of these two datasets would likely influence a bias in the density estimation of Kernel analysis (Harris *et al.* 1990). We also chose concave polygons over convex polygons to ensure that areas not visited by collared individuals were not included in home range calculation; therefore home range size would not be overestimated (Harris *et al.* 1990). We calculated the distance of sleeping trees and feeding trees from the home range boundary, excluding those for individual number 5 for which we had collected data only for one complete nocturnal follow before the signal to its collar was lost.

### Data Analysis

We performed all statistical analysis in IBM SPSS 21.0 (SPSS, Chicago, IL, USA). We used a univariate general linear model to test for significant differences in the usage frequency of tree holes and foliage sleeping sites. The dataset met the assumptions of the linear model, where the response variable was sleeping site type and the test variable was individual uses; we controlled for the differing number of sleeping site records per individual as a random factor. We used Mann–Whitney *U* analysis to test for significant differences in the sleeping site preference between males and females. We used Kruskal–Wallis analysis to test for significant differences in the mean values of each sleeping tree characteristic between individual members of the study population, where the individual was the factor. We transformed sleeping site reuse and sharing rate percentage data to arcsine values and used Mann–Whitney *U* tests to analyze differences in sleeping site characteristics, reuse, and sharing rates between males and females. Additionally, we used Mann–Whitney *U* analysis to test for sex differences in the home range size and distance traveled per night. We chose nonparametric tests to analyze sleeping and ranging data because of our small sample size of mouse lemur individuals; we used one mean value for each measurement per individual to avoid pseudoreplication. We used paired sample *t*-tests to test for significant differences in the distance between the home range boundary to sleeping trees and the distance between the home range boundaries to feeding trees, where one mean value for each individual was used for each category (sleeping tree distance and feeding tree distance). We set an α level of 0.05 to test statistical significance. We analyzed sleeping site distribution and home range boundaries using ArcGIS ArcMap 10.0 (esri, Redlands, CA, USA).

## Ethical Note

All research complied with and adhered to the policies of the University of Bristol and the UK Home Office policies when working with animals. All research adhered to the legal requirements of Madagascar. Research within the Sahamalaza–Iles Radama National Park was permitted by Madagascar National Parks, MNP (Permit number 049/15/MEEF/SG/DGF/DCB.SAP/SCB). The Code of Best Practices for Field Primatology was consulted in the planning of all methods undertaken in this study. Capturing and collaring of the study population individuals took place under the supervision of a professional, experienced veterinarian team; details of the procedure are described in the Collaring section. The authors declare that they have no conflict of interest. The datasets generated and analyzed during the current study are available from the corresponding author upon reasonable request.

## Results

### Sleeping Site Characteristics

We recorded 124 sleeping sites, 30 (24%) of which were used multiple times, giving a total of 171 sleeping site records (105 for males, 66 for females). *Microcebus sambiranensis* used foliage nests significantly more often than tree holes/cavities (general linear model: *F*_1,8_ = 20.767, *P* < 0.001), accounting for 93% (*N* = 159) of sleeping site records, with the remaining 7% (*N* = 12) in tree holes/cavities. There was no significant difference in sleeping site type preference between males and females (Mann–Whitney *U* test: *U* = 29.0, *P* = 0.913). We found 27 tree species used as sleeping sites, of which *Macphersonia gracilis*, *Macarisia lanceolata*, *Sorindeia madagascariensis*, *Diospyros* sp., *Mammea punctata*, and *Ficus tiliaefolia* were used most frequently (Table I).

**Table I:** Tree species used as sleeping sites by *Microcebus sambiranensis* in Anabohazo Forest, northwest Madagascar, from March 28, 2015 to April 26, 2015, with the number of times they were used

Malagasy Name	Scientific name	Family	Frequency used (*N*)
Adabovoara	*Ficus tiliaefolia*	Moraceae	13
Ambarasahy	*Burasaia madagascariensis*	Menispermaceae	2
Fahavalon-kazo	*Clausena inaequalis*	Rutaceae	2
Fanazava	*Turraea sericea*	Menispermaceae	2
Gidroa	*Mascarenhasia arborescens*	Apocynaceae	7
Harongana	*Harongana madagascariensis*	Clusiaceae	8
Hazambo	Unidentified	Unidentified	1
Hazojaoby	*Diospyros* sp*.*	Ebenaceae	21
Hazomahogo	*Scolopia madagascariensis*	Salicaceae	1
Kiropoka	*Margaritaria anomala*	Euphorbiaceae	3
Kisaka	*Brachylaena perrieri*	Asteraceae	1
Korontsana	*Macarisia lanceolata*	Rhizophoraceae	26
Lonjo	*Terminalia perrieri*	Combretaceae	5
Mahabibo	*Anacardium occidentale*	Anacardiaceae	7
Mangarahara	*Trachylobium verrucosum*	Fabaceae	5
Maroampototra	*Macphersonia gracilis*	Sapindanceae	15
Menavony	*Campylospermum anceps*	Ochnaceae	2
Mikonga	*Mimosa* sp*.*	Fabaceae	5
Sambalahy	*Albizia aurisparsa*	Fabaceae	2
Sarin-goavy	Unidentified	Unidentified	2
Selivato	*Grewia boinensis*	Tiliaceae	4
Sely	*Grewia amplifolia*	Tiliaceae	2
Somely	*Broussonetia.*	Moraceae	9
Sondririny	*Sorindeia madagascariensis*	Anacardiaceae	14
Tain-datitra	Unidentified	Unidentified	1
Tampiaka	*Erythroxylum platycladum*	Erythroxylaceae	1
Vahimivoha	*Mammea punctata*	Clusiaceae	10
Total			171

Individuals used 5–12 different species of sleeping tree. The microhabitats surrounding the sleeping trees had a density ranging from 0.13 to 4.04 trees/m^2^ but there was little variation in the mean trees/m^2^ density value among sleeping sites used between individuals (0.39–0.56 trees/m^2^). There was no significant difference in the mean sleeping site microhabitat density between males (0.49 ± 0.23 m^2^) and females (0.48 ± 0.29 m^2^), nor was there a significant difference between members of the study population as a whole for this variable (Table II). The DBH of sleeping trees ranged from 31 to 278 mm with a mean DBH of sleeping trees for each individual from 83 to 112 mm. Individual differences in sleeping tree DBH were not significant (Table II). There was no clear pattern in the height of the sleeping tree or its bole height, which ranged from 2.00 to 11.50 m and 0.50 to 7.00 m, respectively. There was considerable variation in the crown diameter of sleeping site trees, 0.55–7.05 m, but such differences were not significant between males and females (Table II).

**Table II:** Median and range (minimum–maximum) for five sleeping tree characteristics for eight *Microcebus sambiranensis* in Anabohazo forest, northwest Madagascar, March 26, 2015–April 26, 2015, with the results of Kruskal–Wallis tests comparing individuals, and Mann–Whitney *U* tests comparing males and females

Individual	1 (M)	2 (M)	3 (M)	4 (M)	5 (M)	6 (F)	7 (F)	8 (F)	Kruskal–Wallis: inter- individual	Mann–Whitney: between sexes
Micro-habitat density (trees/m^2^)	0.56	0.41	0.54	0.50	0.39	0.47	0.51	0.47	df = 7, *H* = 8.889, *P* = 0.261	*U* = 7.0, *P* = 0.881
	(0.28–2.78)	(0.13–2.15)	(0.20–4.04)	(0.29–2.14)	(0.18–1.36)	(0.26–1.77)	(0.17–1.77)	(0.16–3.66)		
DBH (mm)	87	112	84	93	83	95	107	112	df = 7, *H* = 8.952, *P* = 0.256	*U* = 3.0, *P* = 0.180
	(42–171)	(42–220)	(37–144)	(32–264)	(53–171)	(46–278)	(31–267)	(31–220)		
Tree height (m)	6.56	6.76	6.45	6.76	5.40	5.92	5.20	6.17	df = 7, *H* = 13.626, *P* = 0.058	*U* = 2.0, *P* = 0.101
	(3.50–9.00)	(2.50–10.00)	(2.00–10.00)	(2.00–15.00)	(4.00–9.00)	(3.00–9.50)	(3.50–9.50)	(3.50–15.00)		
Bole height (m)	2.79	2.85	2.76	2.72	2.45	2.90	2.77	2.94	df = 7, *H* = 1.525, *P* = 0.981	*U* = 2.0, *P* = 0.101
	(0.50–4.00)	(0.50–7.00)	(0.50–5.50)	(0.50–6.00)	(1.00–4.50)	(0.50–5.00)	(1.00–5.50)	(1.00–7.00)		
Crown diameter (m)	2.56	3.01	2.14	2.39	2.21	3.15	2.31	2.71	df = 7, *H* = 9.469, *P* = 0.211	*U* = 4.0, *P* = 0.297
	(0.76–5.00)	(0.55–7.05)	(0.86–4.21)	(0.81–6.70)	(1.52–3.03)	(0.61–6.19)	(0.98–5.27)	(0.89–6.10)		
Tree species (*N*)	10	9	11	12	5	12	10	11	/	*U* = 4.5, *P* = 0.362

### Sleeping Site Use

**Fig. 2:**
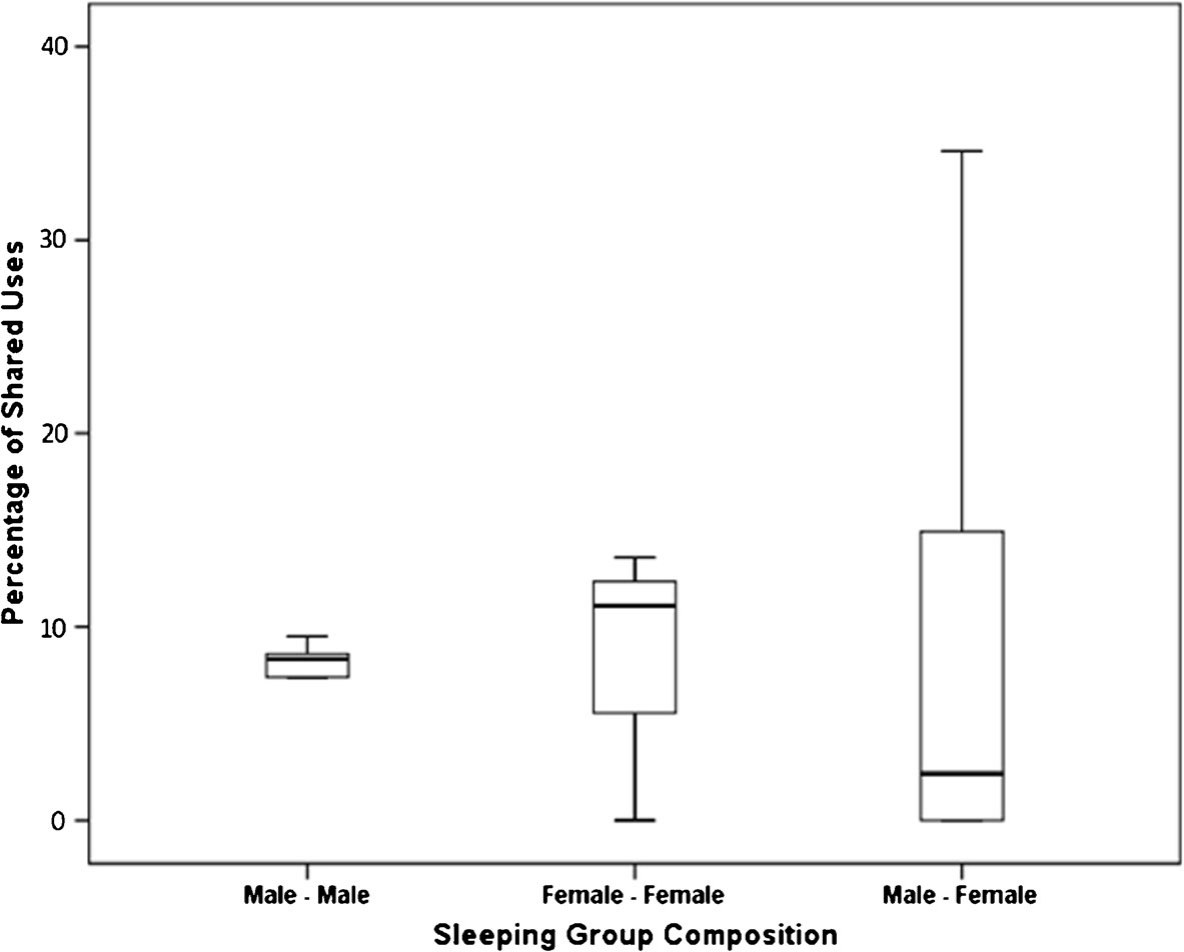
Percentage of sleeping site records that includes shares for eight *Microcebus sambiranensis* in Anabohazo forest, northwest Madagascar, March 26, 2015–April 26, 2015.

We recorded 47 instances of sleeping sites being reused. This represents 27% of the total number of sleeping site uses recorded. Males reused a sleeping site in 19% of sleeping site recordings compared to females that reused a sleeping site in 14% of cases; however, this difference was not significant (Mann–Whitney *U* test: *U* = 4.5, *P* = 0.393). There was no significant difference in the sleeping site sharing rate between males and females (Mann–Whitney *U* test: *U* = 5.5, *P* = 0.571; Fig. 2). Males shared a sleeping site with another collared individual in 15% of recordings. In these instances, the male shared with another male 50% of recordings and with a female 50% of recordings. In comparison, females shared their sleeping site with another collared individual on 21% of recordings; 32% were with another female and 68% were with a male; this bias toward sharing with males is likely due to a higher representation of males within the study population. There were never more than two collared individuals sharing a sleeping site; this is a minimum value as uncollared individuals could not be accounted for. Sleeping groups were not stable and sharing composition of collared individuals varied over time.

## Home Range Size and Nocturnal Ranging

**Fig. 3:**
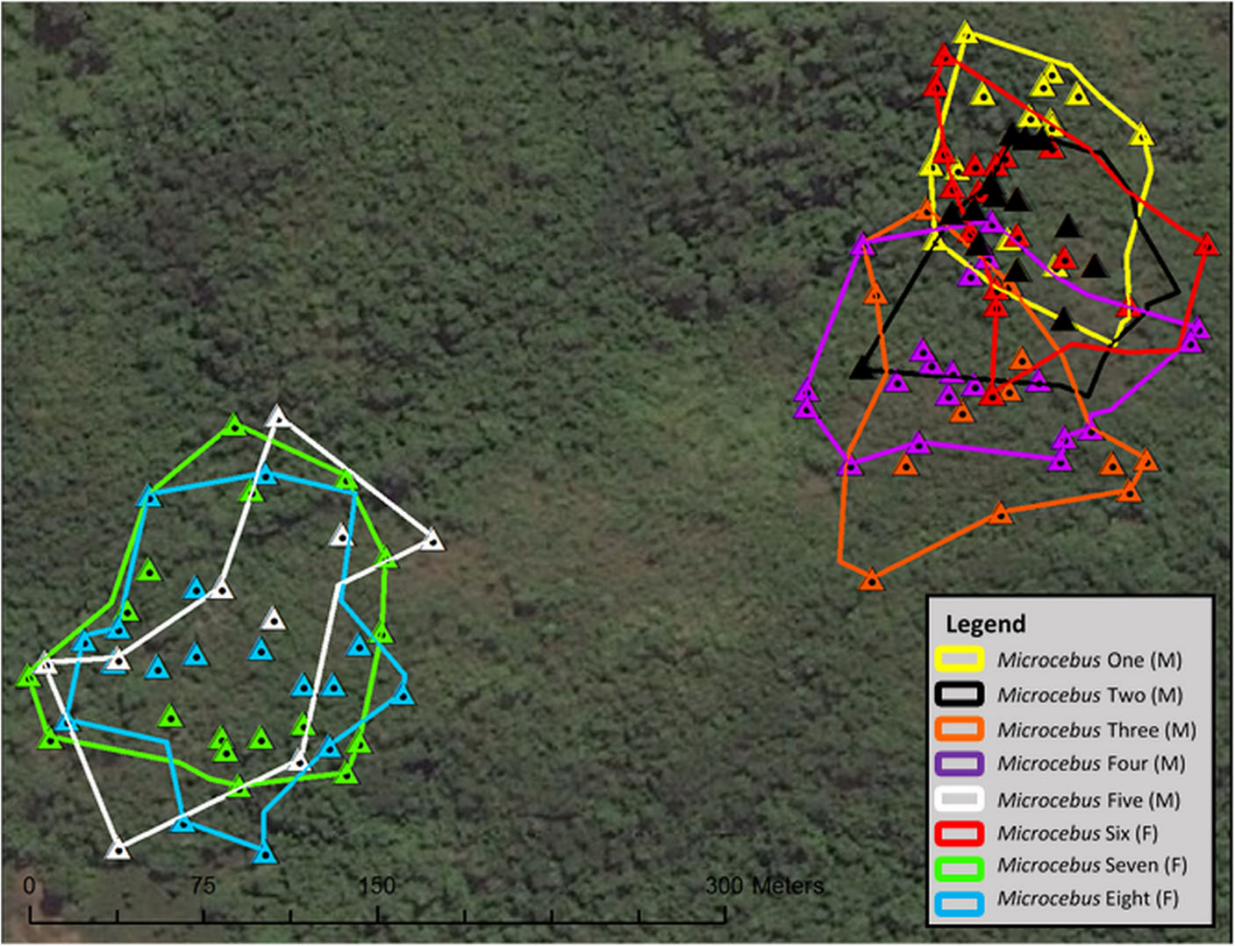
Home ranges of eight collared *Microcebus sambiranensis* individuals in Anabohazo forest, northwest Madagascar, March 26, 2015–May 30, 2015. Triangles indicate sleeping sites. Map created using ESRI ArcGIS ArcMap 10.0, Scale: 1:1700.

The mean size of recorded home ranges was 1.15 ha with a minimum of 0.87 ha and a maximum of 1.61 ha (Table III). Five collared individuals had home ranges that overlapped, forming a neighborhood. The home ranges of the other three individuals also overlapped, forming another neighborhood, separated from the other five individuals by a gap of ca. 180 m (Fig. 3). We sighted a few other *Microcebus sambiranensis* individuals within this gap but they were not collared, nor did we observe them using or leaving a sleeping site used by a member of our collared subpopulation. The mean percentage of overlap between home ranges was 83.53% ± 12.34% with a minimum of 60.17% and a maximum of 100.00% (Table III).

**Table III:** Home range size for eight collared *Microcebus sambiranensis,* with the total overlap with other collared individuals; the percentage overlap by other collared individuals; and the minimum, maximum, and mean distance traveled per night in Anabohazo forest, northwest Madagascar, March 26, 2015–May 30, 2015

Individual (sex)	Area (ha)	Overlap (ha)	Overlap (%)	Number of follows (*N*)	Minimum nightly travel distance (m)	Maximum nightly travel distance (m)	Mean distance traveled per night (m)
1 (M)	0.87	0.64	74.14	5	101	277	230 ± 74
2 (M)	0.90	0.90	100.00	6	140	322	219 ± 73
3 (M)	1.13	0.68	60.17	2	223	246	234 ± 16
4 (M)	1.13	0.91	79.98	6	182	304	240 ± 54
5 (M)	1.23	1.03	83.44	1	129	129	129 ± 0
6 (F)	0.88	0.79	89.66	6	157	274	216 ± 39
7 (F)	1.61	1.41	87.72	5	155	258	191 ± 42
8 (F)	1.41	1.31	93.15	4	153	333	210 ± 83

**Fig. 4:**
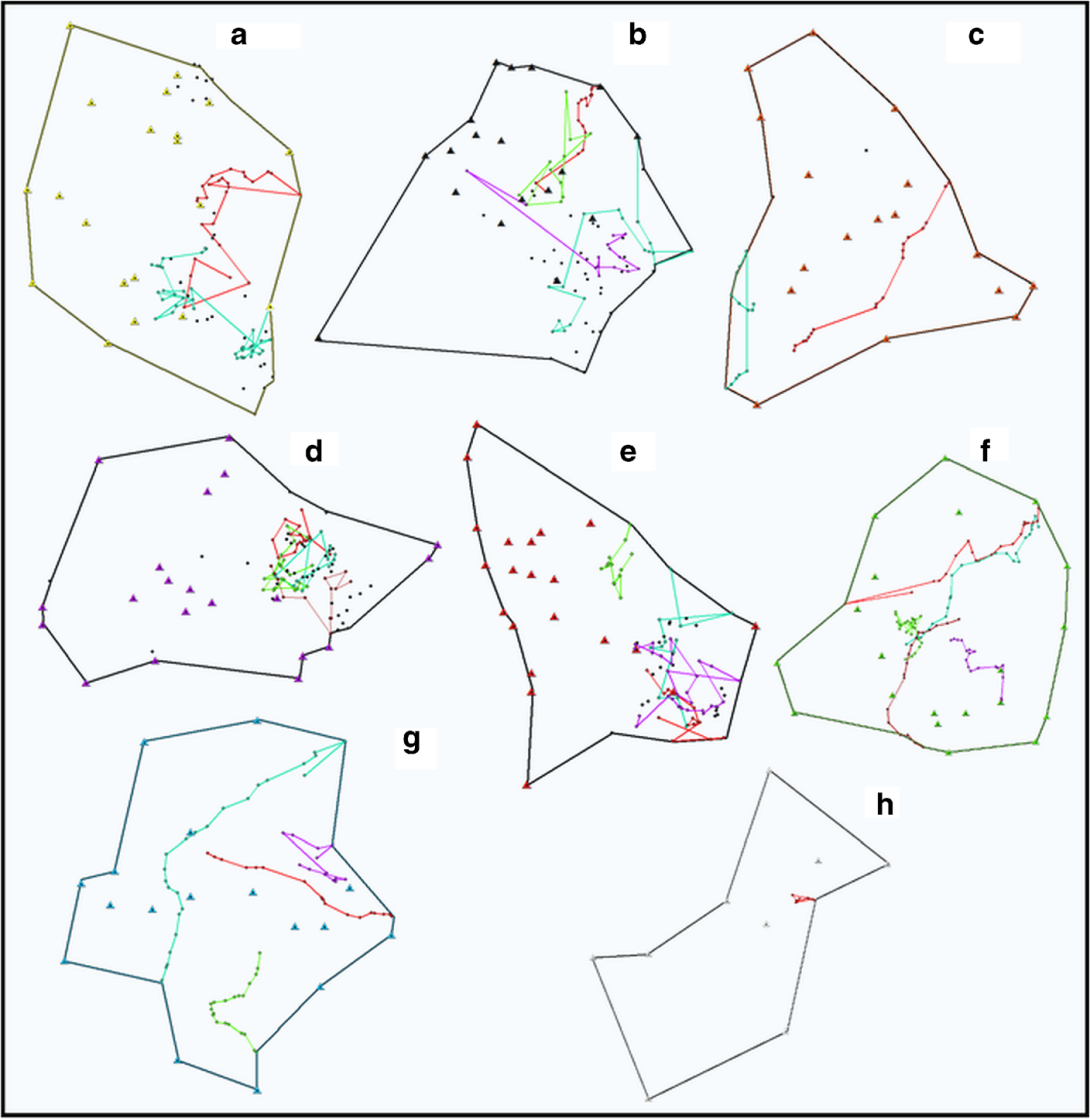
Home ranges of eight collared *Microcebus sambiranensis* individuals in Anabohazo forest, northwest Madagascar with complete night travel routes (*N* = 26) for March 26, 2015–May 30, 2015, including feeding trees indicated by colored lines, sleeping sites indicated by triangles, and additional feeding trees that were not part of a complete nightly follow indicated by black dots. A: *Microcebus* 1, B: *Microcebus* 2, C: *Microcebus* 3, D: *Microcebus* 4, E: *Microcebus* 6, F: *Microcebus* 7, G: *Microcebus* 8. Few data were collected for *Microcebus* 5 (H) so we removed it from analysis.

We recorded 35 nightly travel distances: 20 for males and 15 for females. The mean distance traveled per night was 208 m with a minimum of 101 m and a maximum of 333 m. Of the 35 nocturnal travel distances that we recorded, 26 were complete, 13 for males and 13 for females (Fig. 4).

There was no significant difference between the home range size (Mann–Whitney *U* test: *U* = 4.0, *P* = 0.294), overlap percentage of home ranges (Mann–Whitney *U* test: *U* = 3.0, *P* = 0.180), and distance traveled per night between males and females (Mann–Whitney *U* test: *U* = 123.0, *P* = 0.368).

### Spatial Distribution of Sleeping Sites

Sleeping trees were a mean ± SE distance of 11.0 ± 12.81 m from the home range boundary, with a minimum of 0 m and a maximum of 45 m. In comparison, their feeding trees were located at a mean distance of 21.0 ± 14.09 m from a home range boundary, with a minimum of 0 m and a maximum of 55 m. There was a significant difference between the distance between home range boundaries and sleeping sites compared to the distance between home range boundaries and feeding trees (paired *t*-test: *t* = −3.474, df = 6, *P* = 0.013).

## Discussion

Our results indicate that our study population used a number of different tree species as sleeping sites, and of those species *Macphersonia gracilis*, *Macarisia lanceolata*, *Sorindeia madagascariensis*, *Diospyros* sp., *Mammea punctata*, and *Ficus tiliaefolia* were used the most frequently. This could be due to the high density of their foliage compared to the other tree species, especially in *Macarisia lanceolata* and *Diospyros* sp., which would provide better protection from predators (Aquino and Encarnacion 1986; Rasoloharijaona *et al.* 2003). *Microcebus sambiranensis* did not use tree species such as *Mangifera indica* and *Annona chrysophylla*, which we recorded as feeding trees, as sleeping sites because of their exposed branches and sparse foliage. The individuals in our study population had little variation in the characteristics of the sleeping sites that they used.

In general, sleeping site use and fidelity in *Microcebus sambiranensis* appears to be similar to that of *M. berthae*, with both species having a high usage frequency of foliage nests, alternating sleeping sites regularly, very rarely sharing their sleeping sites, and having unstable sleeping group compositions (Dammhahn and Kappeler 2005; Schwab 2000). The use of foliage nests may be due to restricted availability of suitable tree hole sleeping sites, as suggested for *Microcebus ravelobensis* (Radespiel *et al.* 2003; Thoren *et al.* 2010). The high usage frequency of foliage nests may also be due to competition for tree hole sleeping sites from larger sympatric nocturnal lemur species such as *Lepilemur sahamalazensis* and *Mirza zaza*, both of which are known to use tree holes as sleeping sites (Seiler *et al.* 2013a). The sleeping site choice of *M. sambiranensis* may also be affected by seasonality and they may use tree holes if they are available at certain times of the year, similarly to *L. sahamalazensis; L. sahamalazensis* use tree holes in the dry season, but prefer lianas in the wet season when tree holes are flooded (Seiler *et al.* 2013a). As this investigation took place during the transition between the wet and dry seasons, tree holes may still have been flooded, which could explain our observations of a higher proportion of foliage nest usage compared to tree hole sleeping sites.

The low sleeping site fidelity and unstable sleeping groups of *Microcebus sambiranensis* are similar to behaviors of *M. berthae* and *M. ravelobensis* (Dammhahn and Kappeler 2005; Radespiel *et al.* 2003). In contrast, these behaviors differ from that of *M. murinus*, a species that regularly reuses sleeping sites and sleeps in stable communal groups. These behaviors are suggested to increase the group’s alertness to predators and to provide thermoregulatory benefits (Alexander 1974; Radespiel *et al.* 2003; Schmid 1998). We speculate that *M. sambiranensis* trade off these thermoregulatory benefits while sleeping alone and regularly changing sleeping sites to maintain crypsis and minimize detection by predators (Radespiel *et al.* 2003; Weidt *et al.* 2004) and to reduce the infection risk from transmittable diseases (Nunn and Altizer 2006). Furthermore, the rare instances of communal sleeping in *M. sambiranensis* may be for thermoregulatory benefits to facilitate torpor; we speculate that communal sharing occurs more commonly in the cooler dry season when thermal insulation would be required if *M. sambiranensis* undergoes periods of seasonal torpor (Perret 1998; Radespiel *et al.* 2003; Weidt *et al.* 2004). A study of seasonality in sleeping behavior of *M. sambiranensis* is needed to further investigate sleep sociality of this species.

The home ranges of *Microcebus sambiranensis* overlapped significantly with each other for both males and females, forming social neighborhoods (Clark 1985; Jolly 1966; Richard 1985). Male and female home ranges overlapped extensively, although there was no significant difference in home range size between the sexes. Despite the high degree of home range overlap observed, we collared only eight mouse lemurs and it is possible that additional individuals were in the mouse lemur neighborhoods that were not part of this study. This finding of localized, mixed-sex neighbourhood systems is similar to the ranging ecology observed in other mouse lemur species such as *M. rufus* (Atsalis 2000), *M. murinus* (Radespiel 2000), and *M. berthae* (Dammhahn and Kappeler 2005). This mixed-sex spatial and temporal distribution gives male and female *M. sambiranensis* access to several potential mating partners (Eberle and Kappeler 2004). The spatial distribution of the population of *M. sambiranensis* within a neighborhood social system could suggest a polygynandrous mating system, as in *M. murinus*, *M. rufus*, *M. berthae M. griseorufus*, and *M. ravelobensis* (Atsalis 2000; Eberle and Kappeler 2004; Génin 2008; Radespiel 2000; Weidt *et al.* 2004) and a system where parents and their offspring siblings may live in close proximity to one another and share overlapping home ranges (Dammhahn and Kappeler 2005).

There are few data on the nightly travel distances of mouse lemurs, but *Microcebus berthae* travel up to 4470 m (Dammhahn and Kappeler 2005), a much higher value than we recorded here for *M. sambiranensis*. This difference may be due to different feeding habits in the two species. *M. berthae* has a narrow feeding niche and primarily feeds on invertebrates, such as moths, whereas other mouse lemurs have much broader feeding niches, with a diverse diet including both plant material such as fruit, flowers, nectar, gum, and foliage (Atsalis 1999b; Radespiel 2006), and invertebrate prey (Dammhahn and Kappeler 2008). The higher proportion of stationary food consumed by these other species, including *M. sambiranensis*, means they do not have to travel far to fulfill their dietary needs (Joly and Zimmermann 2007).

We found a large proportion of *Microcebus sambiranensis* sleeping sites near the edge of their home ranges, but they also slept in the center of their ranges. Sleeping trees were located significantly closer to the home range boundary than feeding trees, suggesting that *M. sambiranensis* may sleep peripherally, but forage centrally within their home range, a behavior that has not previously been described for other mouse lemur species. This behavior of remaining close to reliable food sources is well known in diurnal lemurs (Mertl-Millhollen *et al.* 2003), and also occurs in some nocturnal species, such as the sportive lemurs (Pollock 1979). It is possible that *M. sambiranensis* individuals behave in this way to guard their known feeding sites. However, this finding could be an artefact of our analytical approach; we collected sleeping site data on more days than feeding site data, where fewer days were used for nocturnal follows.

*Microcebus sambiranensis* has a small, fragmented geographic distribution over an area of just 700 km^2^ (Mittermeier *et al.* 2010) and understanding the specific use and characteristics of its sleeping sites is vital to its conservation. The number of suitable sleeping trees available is dependent on the quality and management of the surrounding forest habitat (Rasoloharijaona *et al.* 2008). Information on the preferential tree species for sleeping site use by Endangered lemurs is important to conservation non-governmental organizations (NGOs), as it is used to inform their reforestation efforts to increase the proportion of those tree species available within the landscape. Home range data are also valuable to these NGOs as it enables them to perform population viability analysis and to estimate population densities in forest blocks, crucial analyses for the species status assessments carried out by the International Union for Conservation of Nature (IUCN). This study provides these data for *M. sambiranensis* that can now facilitate the conservation of this species, as well as provide autecological data for future investigation and comparisons of behavioral ecology among mouse lemurs.
